# A strain gauge analysis comparing 4‐unit veneered zirconium dioxide implant‐borne fixed dental prosthesis on engaging and non‐engaging abutments before and after torque application

**DOI:** 10.1002/cre2.97

**Published:** 2018-02-15

**Authors:** Alyssa Epprecht, Marco Zeltner, Goran Benic, Mutlu Özcan

**Affiliations:** ^1^ Clinic of Fixed and Removable Prosthodontics and Dental Material Science, Center of Dental Medicine University of Zurich Switzerland

**Keywords:** engaging and non‐engaging abutments, FDP, oral implants, strain gauge, zirconia

## Abstract

This study quantified the strain development after inserting implant‐borne fixed dental prosthesis (FDP) to various implant–abutment joints. Two bone‐level implants (∅ = 4.1 mm, RC, SLA 10 mm, Ti, Straumann) were inserted in polyurethane models (N = 3) in the area of tooth nos 44 and 47. Four‐unit veneered zirconium dioxide FDPs (n = 2) were fabricated, one of which was fixed on engaging (E; RC Variobase, ∅ = 4.5 mm, H = 3.5 mm) and the other on non‐engaging (NE) abutments (RC Variobase, ∅ = 4.5 mm, H = 5.5 mm). One strain gauge was bonded to the occlusal surface of pontic no. 46 on the FDP and the other two on the polyurethane model. Before (baseline) and after torque (35 Ncm), strain values were recorded three times. Data were analyzed using Kruskal–Wallis and Mann–Whitney U tests (α = 0.05). Mean strain values presented significant increase after torque for both E and NE implant–abutment connection type (baseline: E = 4.33 ± 4.38; NE = 4.85 ± 4.85; torque: E = 196.56 ± 188.02; NE = 275.63 ± 407.7; p < .05). Mean strain values based on implant level presented significant increase after torque for both E and NE implant–abutment connection (baseline: E = 4.94 ± 5.29; NE = 5.78 ± 5.69; torque: E = 253.78 ± 178.14; NE = 347.72 ± 493.06; p < .05). The position of the strain gauge on implants (p = .895), FDP (p = .275), and abutment connection type (p = .873) did not significantly affect the strain values. Strain levels for zirconium dioxide implant‐borne FDPs were not affected by the implant–abutment connection type.

## 1 INTRODUCTION

The use of dental implants is a well‐accepted and predictable treatment modality for the rehabilitation of partially or completely edentulous patients (Asvanund, 2014; Hegde, Lemons, Broome, & McCracken, 2009). Although the success rate with implants are high, biological and technical complications around the implants or implant‐borne fixed dental prosthesis (FDP) are reported to increase in long‐span FDPs (Pjetursson, Brägger, Lang, & Zwahlen, 2007). The type of implant–abutment connection, configurations of implant components, or design and biomechanical properties of the FDP material play a significant role on stress distribution around the implants or on the FDP.

Initially, upon tightening, the abutment screw exerts a compressive force to maintain the contact between the abutment and the implant surface (Nishioka, Nishioka, Abreu, de Vasconcellos, & Balducci, 2010). At this moment, the torque applied to the prosthesis‐abutment induces stresses that are transmitted to the supporting bone and suprastructure, which can eventually yield to bone resorption (Asvanund, 2014; Nishioka et al., 2010) or chipping in the veneering ceramic. In fact, mechanical stress may have both positive and negative consequences on the bone tissue (Abreu, Nishioka, Balducci, & Consani, 2012; Isidor, 2006). Although the response to an increased mechanical stress below a certain threshold may increase the bone density or apposition of bone (De Vasconcellos, Özcan, Maziero Volpato, Bottino, & Yener, 2012; Isidor, 2006; Watanabe, Uno, Hata, Neuendorff, & Kirsch, 2000), micro‐damage as a result of mechanical stress beyond the fatigue threshold results in bone resorption (De Vasconcellos et al., 2012, Isidor, 2006, Watanabe et al., 2000).

Typically, engaging (E) abutments are indicated for crowns but can be used for FDPs as the subtle screw holes are more aesthetic than the larger ones as in the case of non‐engaging (NE) abutments. In addition, the height (5.5 mm) of the E abutment is higher, which enables better stability for the framework of the FDP compared to NE (3.5 mm). In practice, the grooves of the E abutments are partially eliminated by grinding manually that could result in more strain development in the bone tissue around the oral implants. However, to date, there is no proof whether E abutments cause more strain development compared to NE abutments in the FDPs, despite the standardized grinding procedures. Moreover, torque forces during tightening of the prosthetic screws also produces compressive forces on the suprastructure (Asvanund, 2014). Depending on the material type, even though the rigid suprastructure appears to fit well on each abutment, residual stresses after the torque may yield to mechanical failures in the FDP (Asvanund, 2014).

Complex strain fields around fixtures, implant components, or suprastructures could be typically measured using strain gauge analysis (Abduo, Bennani, Lyons, Waddell, & Swain, 2011; Abreu et al., 2012; Asvanund, 2014; Castro, Zancope, Verissimo, Soares, & Neves, 2015; Cehreli & Iplikcioglu, 2002; Cho et al., 2014; De Vasconcellos et al., 2012; De Vasconcellos, Nishioka, de Vasconcellos, Balducci, & Kojima, 2013; Heckmann et al., 2014; Hegde et al., 2009; Isidor, 2006; Karl, Rosch, Graef, Talyor, Heckmann, 2015; Karl, Graef, & Wichmann, 2011; Karl, Graef, Wichmann, & Krafft, 2012; Karl & Holst, 2012; Karl & Taylor, 2011; Karl, Wichmann, Heckmann, & Krafft, 2008; Nishioka, de Vasconcellos, & de Melo Nishioka, 2011; Nishioka, de Vasconcellos, Joias, & Rode Sde, 2015). With this method, an electrical resistance in the strain gauge enables the measurement of deformation with high sensitivity (μm/m) (Asvanund, 2014). Strain is defined as the ratio between the length of an object under stress and its original dimension; it is a dimensionless entity. In that respect, a strain gauge is considered an indirect measurement method that analyzes mechanical deformation under physical stress, based on electrical measurements registered with a device called a “transducer” (Nishioka et al., 2010). Because deformations are normally imperceptible to the naked eye, strain gauge is a useful tool as it quantifies a superficial deformation with an electric sensor (Nishioka et al., 2010). The working principle in this method is based on the variation of the electrical resistance transformed into the deformation levels (Nishioka et al., 2010). To the best knowledge of the authors, there is no study to date specifically comparing the strain development in E versus NE abutment types in relation to the FDP type and the torque amount.

The objectives of this study therefore were to quantify the strain development after inserting implant‐borne FDPs to E versus NE abutment types at implant and FDP levels after application of torque on the abutment screw. The null hypothesis tested was that the type of abutment would not influence the strain level at neither implant or FDP levels after application of torque on the abutment screw.

## 2 MATERIALS AND METHODS

### 2.1 Model preparation

Experimental model was fabricated from a phantom model (Nissin Dental Products Ltd, Kyoto, Japan) where the teeth between 43 and 48 were missing, representing a clinical situation requiring implants. In order to simulate the alveolar bone tissue, models (*N* = 3) were poured in polyurethane (Polyurock, Cendres + Métaux SA, Bienne, Switzerland) having similar mechanical properties to the bone.

Two bone‐level implants (∅ = 4.1 mm, RC, SLA 10 mm, Ti, Straumann AG, Basel, Switzerland) were inserted in the polyurethane models in the area of tooth nos 44 and 47 with a 5° angle between the implants. Using impression copings, an impression was made of each model with polyether material (Permadyne, 3 M ESPE, Minn, USA), and the polyurethane models were copied into plaster models. Analog implants (bone level, RC, Implant Analog, L 12 mm, Ti, Straumann AG) were fitted to the impression copings, and a stone cast was made for the fabrication of the FDPs.

### 2.2 Fabrication of the veneered zirconium dioxide FDPs

For each model, six identical 4‐unit zirconium dioxide (Lava, 3 M ESPE, Minn, USA) FDP frameworks were made and were subsequently veneered. Using the master models, the FDPs were designed (Exocad Software, Darmstadt, Germany) and scanned (Ceramill Map 400, Amann Girrbach, Koblach, Austria). Zirconia blocks (Lava) were then milled in a 5‐axis milling machine (Ceramill Motion 2, Amann Girrbach) and sintered (Ceramill therm 3, Amann Girrbach). The zirconia FDPs were tried in and then veneered with feldspathic ceramic (Creation ZI CT, Creation Willi Geller International GmbH, Meiningen, Austria; *N* = 6, *n* = 2 per model) according to the manufacturer's firing instructions.

**Figure 1:**
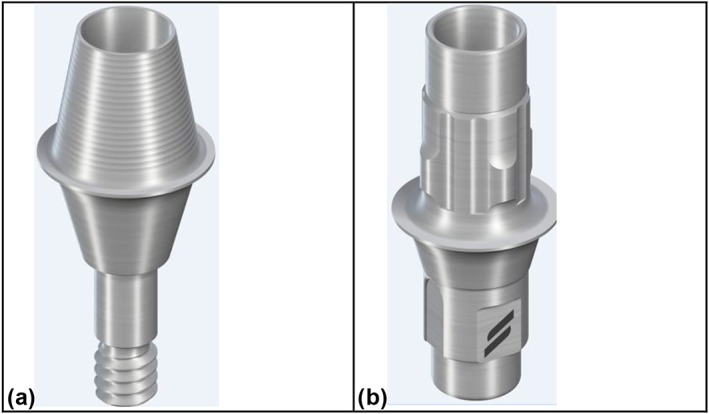
Photos of (a) non‐engaging (*H* = 3.5 mm) and (b) engaging implant–abutment connection (*H* = 5.5 mm)

Nine FDPs were fixed on E (RC Variobase, ø = 4.5 mm, *H* = 3.5 mm, Straumann AG) and NE (RC Variobase, ø = 4.5 mm, Straumann AG) abutments, respectively (Figure 1a,b).

The fixation was accomplished by particle‐abrading the intaglio surfaces of the FDPs and the metal abutments with 50‐μm silica particles coated with Al_2_O_3_ (Rocatec Plus, 3 M ESPE). Both the FDP and the abutments were ultrasonically cleaned (Bransonic Ultrasonic Cleaner 3510, Branson, Danbury, USA) in ethanol for 5 min and dried with oil‐free air. Then, one coat of silane (ESPE‐Sil, 3 M ESPE) was applied on the FDP and the abutment, waited for its reaction for 5 min. Finally, FDPs were cemented on the abutments using chemically polymerized resin cement (Panavia 21, Kuraray GmBH, Tokyo, Japan). The margins of the FDP were coated with the oxygen blocking gel (Oxyguard, Kuraray GmbH) for 5 min. Then, it was washed and dried with oil‐free air.

### 2.3 Strain gauge analysis

**Figure 2:**
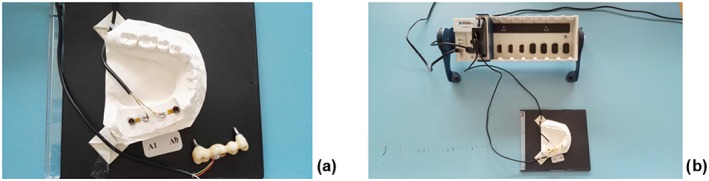
Position of strain gauges (a) placed distally adjacent to implant no. 44, mesially adjacent to implant no. 47 and on occlusal surface of pontic 46 on the fixed dental prosthesis; (b) soldering terminals placed directly next to the strain gauges that are connected to a multichannel bridge amplifier

One strain gauge was bonded to the occlusal surface of the FDP on pontic 46 and the other two on the polyurethane model, one being distal to the 44 implant and the other mesial to 47 implant (Figure 2a,b). In order to position the strain gauges (SGs) precisely on the polyurethane models, a line connecting the two implants was drawn with a ruler and a 0.7‐mm pencil lead. One SG was placed distally adjacent to the implant no. 44 and mesially adjacent to implant no. 47. The third SG was placed on the occlusal surface of pontic no. 46 on the FDP. For exact positioning of SG on the FDP, occlusal surface was made plane. A mesio‐distal line was drawn occlusal, leading exactly through the middle of the pontic 46. The SG was placed on this line, bordering right on the edge of the pontic.

**Figure 3:**
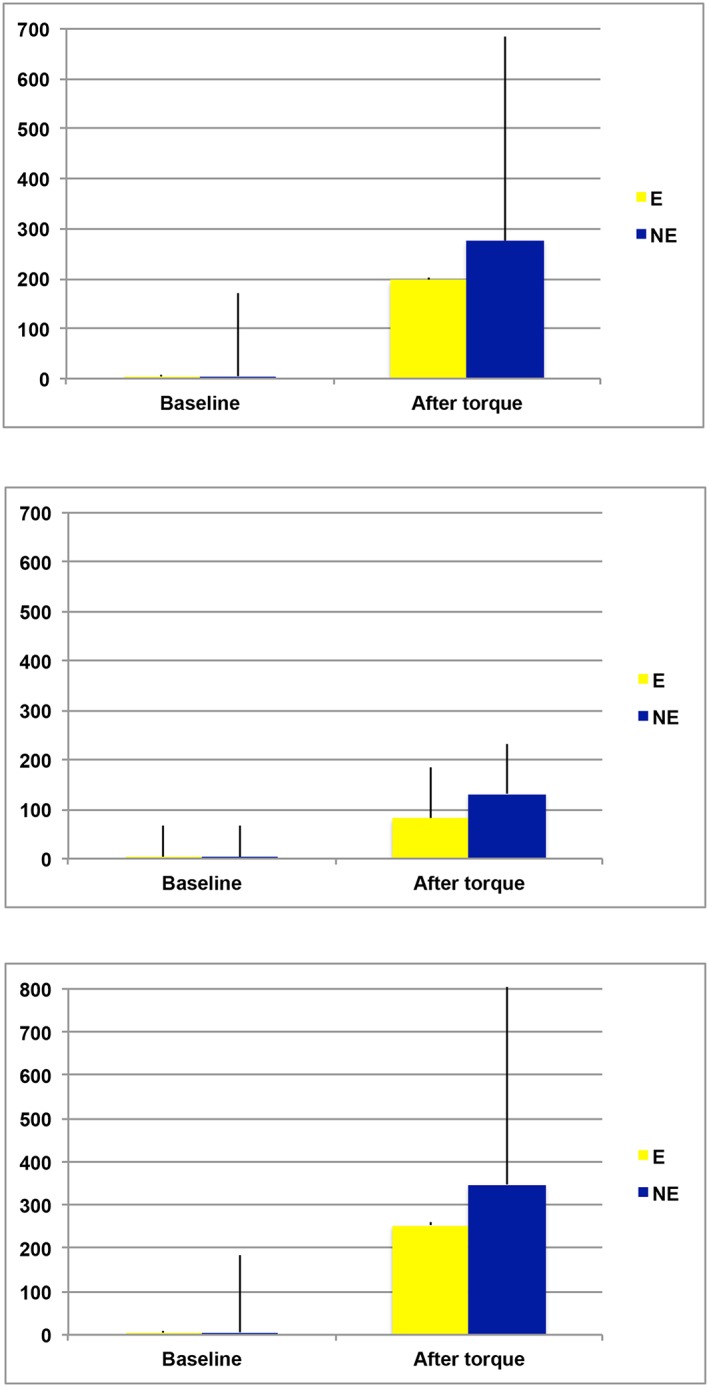
Mean strain values and standard deviations (mV) at baseline and after torque for (a) implant–abutment connection; (b) fixed dental prosthesis 46 occlusal only; (c) implant 44 distal; implant 47 mesial. E = engaging; NE = non‐engaging

The sites were initially cleaned with acetone to ensure good bonding of the SGs. A thin layer of methyl‐2‐cyanacrylate resin (M‐Bond 200; Vishay Measurements Group, Raleigh, NC, USA) was used to fix each SG, which was positioned and held in place under slight pressure for 3 min. Soldering terminals were bonded next to the SGs in the same manner. Each SG was wired separately, and the three SGs were connected to a multichannel bridge amplifier to form one leg of the bridge. A computer was interfaced with the bridge amplifier to record the output signal of the polyurethane and suprastructure surface. Data acquisition system software (SignalExpress, National Instruments, National Instruments Corporation, Austin, TX, USA) was used to record the data.

Each SG was set to zero and calibrated prior to insertion of the FDPs to the implant. Baseline values were noted at 0, 20, and 40 s after calibration. The occlusal screws were tightened onto the abutments until the screw came to a halt. A torque of 35 Ncm was then applied using the manufacturer's manual torque‐controlling device (Straumann AG). Strain was measured again at 0, 20, and 40 s after torque application.

### 2.4 Statistical analysis

Data were analyzed using a statistical software package (SPSS Software V.20, Chicago, IL, USA). Kolmogorov–Smirnov and Shapiro–Wilk tests were used to test normal distribution of the data. As normal distribution was not observed, the data were analyzed using Mann–Whitney *U* test and Kruskal–Wallis nonparametric tests where strain values were the dependent variables and implant position (two levels: 44 vs. 47), abutment type (two levels: E vs. NE), and measurement time (two levels: baseline vs. after torque) were the independent variables. Bonferroni correction was made at *p* < .0083. *p* values less than .05 were considered significant in all tests.

## 3 RESULTS

Because no significant difference was observed between 20 and 40 s (*p* ≥.05), the latter was used in the statistical analysis. Overall, compared to baseline, regardless of the implant and FDP level, mean strain values presented significant increase after torque for both E and NE implant–abutment connection type (baseline: E = 4.33 ± 4.38; NE = 4.85 ± 4.85; torque: E = 196.56 ± 188.02; NE = 275.63 ± 407.7; *p* < .05; Figure 3a).

Considering only the strain values on the FDP level, also, a significant increase was observed for both E and NE (baseline: E = 4E = 3.11 ± 1.9; NE = 3.0 ± 2.31; torque: E = 82.11 ± 64.22; NE = 131.44 ± 101.74; *p* < .05; Figure 3b).

Mean strain values based on implant level presented significant increase after torque for both E and NE implant–abutment connection type (baseline: E = 4.94 ± 5.29; NE = 5.78 ± 5.69; torque: E = 253.78 ± 178.14; NE = 347.72 ± 493.06; *p* < .05; Figure 3c).

There was no statically significant difference with regard to strain adjacent to the implants (*p* = .895) and the FDP (*p* = .275) or between the implant strain and FDP strain (*p* = .873).

Strain levels for 4‐unit veneered zirconium dioxide implant‐borne FDPs were not affected by the implant–abutment connection type on the model tested.

## 4 DISCUSSION

This study was undertaken in order to evaluate the strain development after inserting implant‐borne zirconia FDPs to E versus NE abutment types at implant and FDP level before and after torque application. On the basis of the results obtained, because abutment types and position of the implants did not significantly affect the strain development, the first part of the null hypothesis could be accepted. After torque application, however, strain values increased significantly in all conditions. Thus, the null hypothesis on torque effect could be rejected.

A number of factors might influence strain development at the implant and FDP level such as the effect of axial and non‐axial loading (Cho et al., 2014; Nishioka et al., 2010; Nishioka et al., 2011), straight and offset implant placement (Cho et al., 2014; Nishioka et al., 2010), impression technique, fabrication method of the FDPs, retention type and ceramic veneering (Cehreli & Iplikcioglu, 2002; Karl & Taylor, 2011), the type of implant–abutment joint (Cho et al., 2014; Hegde et al., 2009; Karl et al., 2008), and the type of prosthetic coping (Abreu et al., 2012). Because no data are available in the current literature focusing on the difference in strain development between E and NE abutments, a direct comparison with the other studies would not be possible. Yet, in general, it is commonly accepted and widely reported that after torque application, strain levels increase at both implant and FDP level (Nishioka et al., 2010).

Principally, the cervical region of the implant is the site where the highest stresses occur (Karl & Holst, 2012; Watanabe et al., 2000). This phenomenon is due to the fact that when two materials are in contact with each other and one of them is loaded, the stresses will be higher at the first point of contact in any material (Nishioka et al., 2015). Therefore, the cervical region of the implant is the site where the greatest microdeformations occur, regardless of the type of bone, the design of the implant, the configuration of the prosthesis, and the load (Karl & Holst, 2012, Watanabe et al., 2000). Hence, in this study, the SGs were bonded adjacent to the implant on the polyurethane block through which strain development has been measured by means of SGs. The model could not allow positioning the SGs at the buccal aspect of the implants due to non‐flat surfaces. A similar manner of SG positioning was practiced in previous studies, noting that the models were obtained from a real patient case. A standardized model could have allowed such a positioning. Nevertheless, in multiple unit FDPs, deflection occurs mesial and distal of the abutments during loading (Karl & Holst, 2012, Watanabe et al., 2000). Thus, strain values from the regions are of clinical relevance. Moreover, technically, these regions presented completely flat surfaces enabling accurate bonding of the SGs.

In an attempt to simulate the alveolar bone, polyurethane blocks were used in this study similar to numerous previous studies (Abreu et al., 2012; Cho et al., 2014; De Vasconcellos et al., 2012; Karl et al., 2008; Nishioka et al., 2015; Watanabe et al., 2000). Even though the use of this material is common practice in strain analysis in implantology, polyurethane is assumed to be linearly elastic and isotropic, meaning that the material has the same mechanical properties in all direction. In turn, bone is anisotropic and contains voids, and quality of the bone varies as a function of many other factors (Karl et al., 2008). Nevertheless, the polyurethane model eliminates possible confounding factors related to the biological bone substance.

The results of the current study presented no significant difference in strain development between E and NE implant–abutment connections. It has to be however noted that after inserting the FDPs in the polyurethane models and applying a torque of 35 Ncm, more strain development was observed in the NE abutments compared to the E ones. Yet the results were not significant. This is most probably due to the limited sample size, and therefore, this study should be considered as a pilot one. Certainly, impression methods and the duplication procedures of multiple models add to the misfit of the FDP (Karl et al., 2008). Similarly, the transfer of implant position from polyurethane to plaster models could increase errors that eventually affect the accuracy of the measurement between each model. However, this inherent error is valid for both the implant and the FDP and maybe less in a clinical scenario where single duplication is needed.

Nonetheless, the E abutments contain insertion grooves in order to avoid undesired rotational movement within the implant, whereas the NE one does not. Insertion grooves are beneficial elements in E abutments in order to avoid rotation of single crowns, whereas this becomes less of an issue in an FDP. On the other hand, even though the favorable height of E abutments for crowns may be also useful for the FDPs compared to NE abutments, unfortunately, insertion grooves on E abutments do not allow easy path of insertion for the FDPs, and thus, they were adjusted manually using rotating instruments. One reason for the increased tendency for high strain formation with the NE ones, which are typically indicated for crowns as opposed to NE ones, being indicated for FDPs, could be due to the configuration differences, namely, NE implant–abutment connections present a larger screw hole but less height than that of E ones. Hence, it can be anticipated that less height of the NE abutment did not support the framework and the veneering ceramic compared to E one, and consequently after torque, unsupported areas in the FDP caused more stress and thereby more strain development with this abutment both in FDP and implant level.

The results of this study should be verified in a larger sample, noting that they are costly studies. This pilot study allowed us to calculate the power for similar future studies in that 26 specimens are needed with relevant difference of 80 mV and standard deviation of 100 mV between groups at 80% certainty based on two‐sided two‐sampled *t*‐test.

## 5 CONCLUSIONS

E or NE abutments presented similar strain development at both implant and FDP level after torque application compared to baseline measurements. Strain levels at the implant level were higher than on the FDP yet being not significant. Both E and NE abutments could be advised in conjunction with 4‐unit veneered zirconia FDPs as they demonstrated similar increase in strain development after torque application.

## CONFLICT OF INTEREST

The authors declare that they have no conflict of interests related to this study.

## DISCLOSURE OF INTERESTS

The authors claim to have no financial interest, either directly or indirectly, in the products or information listed in the paper.

## References

[cre297-bib-0001] Abduo, J. , Bennani, V. , Lyons, K. , Waddell, N. , & Swain, M. (2011). A novel in vitro approach to assess the fit of implant frameworks. Clinical Oral Implants Research, 22, 658–663. 21044168 10.1111/j.1600-0501.2010.02019.x

[cre297-bib-0002] Abreu, C. W. , Nishioka, R. S. , Balducci, I. , & Consani, R. L. (2012). Straight and offset implant placement under axial and nonaxial loads in implant‐supported prostheses: Strain gauge analysis. Journal of Prosthodontics, 21, 535–539. 22905920 10.1111/j.1532-849X.2012.00871.x

[cre297-bib-0003] Asvanund, P. (2014). A strain gauge analysis comparing external and internal implant‐abutment connections. Implant Dentistry, 23, 206–211. 24614880 10.1097/ID.0000000000000063

[cre297-bib-0004] Castro, C. G. , Zancope, K. , Verissimo, C. , Soares, C. J. , & Neves, F. D. (2015). Strain analysis of different diameter Morse taper implants under overloading compressive conditions. Braz Oral Res (Epub).. 10.1590/1807-3107BOR-2015.vol29.0028 25627892

[cre297-bib-0005] Cehreli, M. C. , & Iplikcioglu, H. (2002). In vitro strain gauge analysis of axial and off‐axial loading on implant supported fixed partial dentures. Implant Dentistry, 11, 286–292. 12271568

[cre297-bib-0006] Cho, Y. E. , Park, E. J. , Koak, J. Y. , Kim, S. K. , Heo, S. J. , & Park, J. M. (2014). Strain gauge analysis of occlusal forces on implant prostheses at various occlusal heights. The International Journal of Oral & Maxillofacial Implants, 29, 1034–1041. 25216126 10.11607/jomi.3040

[cre297-bib-0007] De Vasconcellos, D. K. , Özcan, M. , Maziero Volpato, C. Â. , Bottino, M. A. , & Yener, E. S. (2012). Strain gauge analysis of the effect of porcelain firing simulation on the prosthetic misfit of implant‐supported frameworks. Implant Dentistry, 21, 225–229. 22614845 10.1097/ID.0b013e3182566e59

[cre297-bib-0008] De Vasconcellos, L. G. , Nishioka, R. S. , de Vasconcellos, L. M. , Balducci, I. , & Kojima, A. N. (2013). Microstrain around dental implants supporting fixed partial prostheses under axial and non‐axial loading conditions, in vitro strain gauge analysis. The Journal of Craniofacial Surgery, 24, e546–e551. 24220463 10.1097/SCS.0b013e31829ac83d

[cre297-bib-0009] Heckmann, S. M. , Karl, M. , Wichmann, M. G. , Winter, W. , Graef, F. , & Taylor, T. D. (2014). Cement fixation and screw retention: Parameters of passive fit. An in vitro study of three‐unit implant‐supported fixed partial dentures. Clinical Oral Implants Research, 15, 466–473. 10.1111/j.1600-0501.2004.01027.x 15248882

[cre297-bib-0010] Hegde, R. , Lemons, J. E. , Broome, J. C. , & McCracken, M. S. (2009). Validation of strain gauges as a method of measuring precision of fit of implant bars. Implant Dentistry, 18, 151–161. 19359866 10.1097/ID.0b013e318192e246

[cre297-bib-0011] Isidor, F. (2006). Influence of forces on peri‐implant bone. Clinical Oral Implants Research, 17, 8–18. 10.1111/j.1600-0501.2006.01360.x 16968378

[cre297-bib-0012] Karl, M. , Graef, F. , & Wichmann, M. (2011). Strain development of implant‐supported fixed prostheses copy milled from zirconia ceramic. The International Journal of Prosthodontics, 24, 479–481. 21909492

[cre297-bib-0013] Karl, M. , Graef, F. , Wichmann, M. , & Krafft, T. (2012). Passivity of fit of CAD/CAM and copy‐milled frameworks, veneered frameworks, and anatomically contoured, zirconia ceramic, implant‐supported fixed prostheses. The Journal of Prosthetic Dentistry, 107, 232–238. 22475466 10.1016/S0022-3913(12)60067-5

[cre297-bib-0014] Karl, M. , & Holst, S. (2012). Strain development of screw‐retained implant‐supported fixed restorations: Procera implant bridge versus conventionally cast restorations. The International Journal of Prosthodontics, 25, 166–169. 22371839

[cre297-bib-0015] Karl, M. , Rosch, S. , Graef, F. , Talyor, T. D. , & Heckmann, S. M. (2015). Strain situation after fixation of three‐unit ceramic veneered implant superstructures. Implant Dentistry, 14, 157–165. 10.1097/01.id.0000163809.37466.ac 15968188

[cre297-bib-0016] Karl, M. , & Taylor, T. D. (2011). Effect of material selection on the passivity of fit of implant‐supported restorations created with computer‐aided design/computer‐assisted manufacture. The International Journal of Oral & Maxillofacial Implants, 26, 739–745. 21841982

[cre297-bib-0017] Karl, M. , Wichmann, M. G. , Heckmann, S. M. , & Krafft, T. (2008). Strain development in 3‐unit implant‐supported CAD/CAM restorations. The International Journal of Oral & Maxillofacial Implants, 23, 648–652. 18807560

[cre297-bib-0018] Nishioka, R. S. , de Vasconcellos, L. G. , & de Melo Nishioka, G. N. (2011). Comparative strain gauge analysis of external and internal hexagon, Morse taper, and influence of straight and offset implant configuration. Implant Dentistry, 20, e24–e32. 21448016 10.1097/ID.0b013e318211fce8

[cre297-bib-0019] Nishioka, R. S. , de Vasconcellos, L. G. , Joias, R. P. , & Rode Sde, M. (2015). Load‐application devices: A comparative strain gauge analysis. Brazilian Dental Journal, 26, 258–262. 26200149 10.1590/0103-6440201300321

[cre297-bib-0020] Nishioka, R. S. , Nishioka, L. N. , Abreu, C. W. , de Vasconcellos, L. G. , & Balducci, I. (2010). Machined and plastic copings in three‐element prostheses with different types of implant‐abutment joints: A strain gauge comparative analysis. Journal of Applied Oral Science, 18, 225–230. 20856998 10.1590/S1678-77572010000300005 PMC5349059

[cre297-bib-0021] Pjetursson, B. E. , Brägger, U. , Lang, N. P. , & Zwahlen, M. (2007). Comparison of survival and complication rates of tooth‐supported fixed dental prostheses (FDPs) and implant‐supported FDPs and single crowns (SCs). Clinical Oral Implants Research, 18, 97–113. 17594374 10.1111/j.1600-0501.2007.01439.x

[cre297-bib-0022] Watanabe, F. , Uno, I. , Hata, Y. , Neuendorff, G. , & Kirsch, A. (2000). Analysis of stress distribution in a screw‐retained implant prosthesis. The International Journal of Oral & Maxillofacial Implants, 15, 209–218. 10795453

